# Amino Acid Metabolism is Significantly Altered at the Time of Admission in Hospital for Severe COVID-19 Patients: Findings from Longitudinal Targeted Metabolomics Analysis

**DOI:** 10.1128/spectrum.00338-21

**Published:** 2021-12-08

**Authors:** Laura Ansone, Monta Briviba, Ivars Silamikelis, Anna Terentjeva, Ingus Perkons, Liga Birzniece, Vita Rovite, Baiba Rozentale, Ludmila Viksna, Oksana Kolesova, Kristaps Klavins, Janis Klovins

**Affiliations:** a Latvian Biomedical Research and Study Centre, Riga, Latvia; b Riga Stradins University, Riga, Latvia; c Institute of Food Safety, Animal Health and Environment (BIOR), Riga, Latvia; d Riga Technical University, Riga, Latvia; Karolinska Institutet

**Keywords:** COVID-19, SARS-CoV-2, longitudinal study, metabolomics, virus-host interactions

## Abstract

The heterogeneity in severity and outcome of COVID-19 cases points out the urgent need for early molecular characterization of patients followed by risk-stratified care. The main objective of this study was to evaluate the fluctuations of serum metabolomic profiles of COVID-19 patients with severe illness during the different disease stages in a longitudinal manner. We demonstrate a distinct metabolomic signature in serum samples of 32 hospitalized patients at the acute phase compared to the recovery period, suggesting the tryptophan (tryptophan, kynurenine, and 3-hydroxy-DL-kynurenine) and arginine (citrulline and ornithine) metabolism as contributing pathways in the immune response to SARS-CoV-2 with a potential link to the clinical severity of the disease. In addition, we suggest that glutamine deprivation may further result in inhibited M2 macrophage polarization as a complementary process, and highlight the contribution of phenylalanine and tyrosine in the molecular mechanisms underlying the severe course of the infection. In conclusion, our results provide several functional metabolic markers for disease progression and severe outcome with potential clinical application.

**IMPORTANCE** Although the host defense mechanisms against SARS-CoV-2 infection are still poorly described, they are of central importance in shaping the course of the disease and the possible outcome. Metabolomic profiling may complement the lacking knowledge of the molecular mechanisms underlying clinical manifestations and pathogenesis of COVID-19. Moreover, early identification of metabolomics-based biomarker signatures is proved to serve as an effective approach for the prediction of disease outcome. Here we provide the list of metabolites describing the severe, acute phase of the infection and bring the evidence of crucial metabolic pathways linked to aggressive immune responses. Finally, we suggest metabolomic phenotyping as a promising method for developing personalized care strategies in COVID-19 patients.

## OBSERVATION

More than a year has passed since the World Health Organization (WHO) announced the COVID-19 outbreak as a pandemic in March 2020, following the rapid spread of the SARS-CoV-2 virus (1). The clinical course of COVID-19 is versatile; the infection of the SARS-CoV-2 virus not only varies in its severity from asymptomatic or mild and moderate respiratory disease (80%) to clinically severe or critical life-threatening disease (20%) but also varies in a range of organs the disease can affect (2, 3). Diverse clinical trajectories seem to be the result of the immune response differences between individuals (4). Multiple innate and adaptive immune system pathways that produce inflammatory molecules against the virus and virus-infected human cells are triggered after the SARS-CoV-2 entry in the cell, with characteristic overexpression of proinflammatory cytokines (e.g., IL-6, TNF-α, IFN-γ) known as cytokine storm in the most severe cases (4–6). The host's immune responses typically involve changes in metabolic processes at the cellular level, reflecting the host-defense mediators and underlying mechanisms (7).

Detailed understanding of the molecular mechanisms behind COVID-19 pathogenesis and inflammatory response is needed to predict and reduce individual risks, develop therapeutic strategies, and reduce the overall mortality rate (around 2% globally according to WHO data) (8). The human blood sera metabolome (defined as small molecules <1500–2000 Da) reflects the organism's metabolic state and is widely used to gain a deeper understanding of the pathogenesis of diseases. Recent reports of metabolomics studies highlight the pivotal role of cellular metabolites in programming immune response to SARS-CoV-2 infection, but nevertheless, none of the studies so far have addressed the metabolomic changes during the recovery of infection in a longitudinal manner (9–13). Considering the extremely high heterogeneity of the COVID-19 disease and lack of promising predictive biomarkers, we believe that implications of longitudinal metabolite profiling may be beneficial in understanding the underlying mechanisms of the diverse course of the disease and promote the early identification of people at increased risk of severe illness from COVID-19 and related complications.

We performed quantitative targeted metabolome analysis with liquid chromatography-mass spectrometry (LC-MS) in blood sera of 32 hospitalized COVID-19 patients at the acute phase (time of admission at the hospital) and the recovery phase (40 ± 14.92 days) of the disease (see Text S1 in the supplemental material for a detailed description of methods). We also included a group of 39 subjects without any acute infection or state from the general population as controls. Written informed consent was obtained from every participant before their inclusion in the study, and the study protocol was approved by the Central Medical Ethics Committee of Latvia (No. 01-29.1.2/928).

As expected, the clinical blood tests revealed abnormal hematological parameters for the majority of study participants at the time of hospitalization, with a high variation in platelet (202.94 ± 65.26 μL) levels and low lymphocyte measurements (0.64 ± 0.56 μL), which coincides with previously reported lymphopenia as the hallmark of severe COVID-19 cases. We also observed a high variation of several markers (e.g., alanine aminotransferase, bilirubin, lactate dehydrogenase, C-reactive protein) indicating renal and hepatic dysfunction, myocarditis, inflammation, and coagulation, which confirms the systemic response to the infection in our study cohort (2, 14) (see Table S1 in the supplemental material).

**FIG 1:**
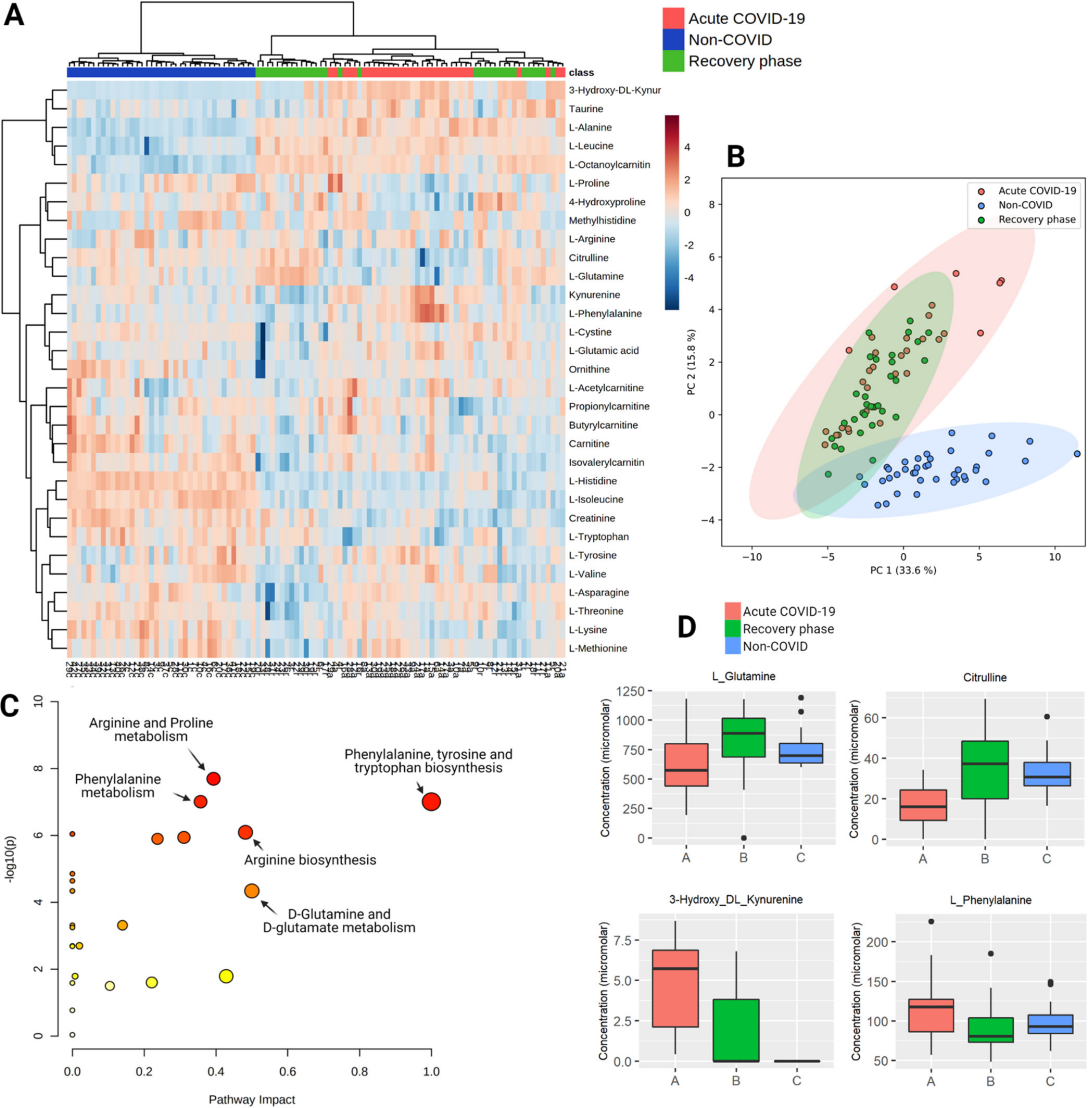
Targeted metabolomic analysis of longitudinal serum samples of hospitalized COVID-19 patients. (A) Heatmap and hierarchical clustering of top 22 significantly altered metabolites. Each column represents one sample: red, samples collected in the acute phase; green, samples collected during the recovery phase; blue, samples collected from the population controls. Each row conforms to a specific metabolite expressed in normalized, log-transformed concentration value. (B) Principal-component analysis showing clear discrimination of samples collected from COVID-19 patients in the acute (red) and recovery (green) phases of infection as well as control subjects (blue) based on the obtained metabolite profiles. (C) Scatterplot representing the most relevant metabolic pathways from KEGG library arranged by adjusted *P* values (obtained by Global Test pathway enrichment analysis) on the *y*-axis, and pathway impact values (from pathway topology analysis) on the *x*-axis. The node color is based on its *P* value and the node radius is determined based on their pathway impact values. (D) Boxplots showing the normalized levels of the most functionally relevant metabolites altered during the recovery of COVID-19 (red, acute phase; green, recovery phase; blue, controls), described as the minimum value, the first quartile, the median, the third quartile, and the maximum value, with the black dots representing outliers.

Out of 51 metabolites analyzed by LC-MS, 22 metabolites showed significantly altered levels (paired *t* test, FDR < 0.05) in the serum samples during the acute phase in comparison to the recovery phase (Table 1), where concentrations for 16 compounds were significantly elevated, whereas 8 metabolites were decreased. The hierarchical clustering and principal-component analysis of the obtained metabolomic profiles showed clear metabolomics-based discrimination of samples collected in different phases of the disease and independent controls (Fig. 1A and B), indicating an altered metabolic activity during infection. Pathway analysis of longitudinally obtained COVID-19 patients’ metabolite profiles revealed 13 significantly enriched pathways (FDR < 0.05), including phenylalanine, tyrosine, and tryptophan biosynthesis, d-glutamine and d-glutamate metabolism, and arginine biosynthesis (Fig. 1C, Table S3). Statistical analysis was done with Metaboanalyst version 5.0 (15).

**TABLE 1:** Serum metabolites showing significantly altered levels between the acute phase and recovery phase of the disease^*a*^

Compound	Fold change	False discovery rate	Avg level in acute COVID-19 μM (±SD)	Avg level in COVID-19 recovery phase μM (±SD)	Avg level in non-COVID controls μM (±SD)
3-Hydroxy-DL-Kynurenine	10.51	6.79E-07	4.77 (± 2.63)*	**1.76 (± 2.59)**	<LOD
4-Hydroxyproline	0.42	1.99E-06	6.4 (± 3.51)*	**15.08 (± 9.34)**	11.24 (± 5.29)
Carnitine	1.25	8.90E-05	**52.74 (± 19.33)**	**42.52 (± 11.56)**	74.52 (± 24.65)
Citrulline	0.48	9.16E-08	16.81 (± 8.48)*	**34.54 (± 17.31)**	32.43 (± 9.66)
Isovalerylcarnitine	2.01	1.78E-06	**0.13 (± 0.08)**	**0.07 (± 0.03)**	0.18 (± 0.09)
Kynurenine	1.71	2.67E-06	3.57 (± 1.40)*	**2.22 (± 1.32)**	2.84 (± 0.69)
L-Acetylcarnitine	1.21	2.34E-02	4.53 (± 2.40)*	3.61 (± 1.26)	4.19 (± 2.75)
L-Asparagine	1.51	3.88E-05	42.56 (± 14.92)	**30.19 (± 11.12)**	44.63 (± 14.64)
L-Glutamic acid	1.65	4.61E-05	174.32 (± 81.10)*	122.38 (± 83.16)	144.62 (± 75.42)
L-Glutamine	0.72	5.71E-08	608.72 (±223.93)*	**820.83 (±268.53)**	732.84 (±131.12)
L-Isoleucine	1.21	1.25E-02	**75.72 (± 18.49)**	**71.38 (± 33.53)**	185.44 (± 71.27)
L-Lysine	1.08	1.15E-02	**174.3 (± 54.09)**	**165.07 (± 32.74)**	225.46 (± 63.36)
L-Methionine	1.47	1.62E-06	**25.99 (± 10.49)**	**17.42 (± 5.79)**	29.69 (± 12.20)
L-Octanoylcarnitine	0.75	3.85E-02	**0.32 (± 0.19)**	**0.42 (± 0.17)**	0.06 (± 0.06)
L-Phenylalanine	1.33	6.79E-07	113.06 (± 38.07)*	88.98 (± 28.46)	95.81 (± 20.50)
L-Proline	0.86	3.23E-03	185.87 (± 81.55)*	219.66 (± 66.13)	236.84 (± 70.70)
L-Threonine	1.45	2.15E-03	**119.92 (± 45.29)**	85.33 (± 37.11)	149.53 (± 40.05)
L-Tryptophan	0.83	2.87E-04	63.23 (± 20.67)	**77.02 (± 16.74)**	93.21 (± 21.87)
L-Tyrosine	1.14	2.87E-04	76.46 (± 21.50)	**70.2 (± 17.15)**	82.52 (± 23.17)
L-Valine	1.26	7.73E-05	254.27 (± 73.92)	**210.84 (± 56.92)**	285.98 (± 83.58)
Ornithine	1.37	9.45E-03	**93.43 (± 30.49)**	**74.36 (± 36.16)**	131.28 (± 72.24)
Taurine	1.36	6.01E-03	108.82 (± 51.77)*	85.87 (± 54.56)	66.89 (± 21.32)

a Only serum metabolites exhibiting significantly altered levels comparing measures obtained during the acute phase and recovery phase of the disease are shown with the corresponding fold change and false discovery rate values obtained from *t* test analysis. Average levels in the acute or recovery phase that differ significantly from the control group are displayed in bold. Asterisk indicates significantly different levels comparing samples from the acute COVID-19 group versus the control group, for which the direction of change is the same as the acute vs recovery group. LOD, level of detection; SD, standard deviation.

We found l-glutamine (Fig. 1D) as the most significantly changed amino acid between the paired patients’ serum samples with reduced acute phase concentrations. l-glutamine levels were also significantly lower in population controls. It is known that glutamine deprivation and decreased glutaminolysis inhibits M2 macrophage polarization, which may partly explain the hyperinflammatory state in severe COVID-19 cases (16, 17). Moreover, the beneficial effect of glutamine has been proposed in multiple studies, where adding enteral l-glutamine to the regular nutrition shortened the duration of hospitalization and improved the outcome in moderate to severe COVID-19 cases (18, 19).

Two other amino acids involved in arginine catabolism through the Urea cycle, citrulline (Fig. 1D) and ornithine (Table 1), were found to significantly change between the acute and recovery phases. However, alterations in the levels of l-arginine itself were not detected. Low blood plasma citrulline levels have already been reported in COVID-19 patients, whereas in patients with severe sepsis, the decreased citrulline levels are associated with acute respiratory distress syndrome (11, 13, 20). Since arginine can be metabolized to creatine and then to creatinine, both varying highly in our study cohort according to regular blood tests performed during hospitalization, arginine catabolism may be implicated in disturbed kidney function of COVID-19 patients (21).

Three out of 22 metabolites showing significantly changed levels between acute infection and recovery phase are involved in the tryptophan-kynurenine pathway: l-tryptophan, kynurenine, and 3-hydroxy-DL-kynurenine (Fig. 1D, Table 1). 3-hydroxy-DL-kynurenine was the marker with the most pronounced fold change between the acute and recovery phases and remarkably was below the detection limit in any of the control samples. The reduction of tryptophan levels and increase of kynurenine and 3-hydroxy-DL-kynurenine in the acute phase supports the conclusions of previous reports and confirms this pathway's key role in severe COVID-19 cases (10, 11). *In vitro* experiments have shown that tryptophan deprivation sensitizes T cells to apoptosis, inhibits proliferation of T cells, and plays a role in CD8 T-cell suppression in cancer (22, 23). Notably, the tryptophan-kynurenine pathway shows a modulatory effect on the macrophage-mediated responses by targeting the synthesis of the metabolic coenzyme NAD+ (24, 25). According to Thomas et al., dysregulated tryptophan metabolism, an essential regulator of inflammation and immunity, may be a potential explanation for severity in older COVID-19 patients (11).

Finally, we observed a significant difference in l-phenylalanine (Fig. 1D) and tyrosine levels in our cohort between the analyzed disease phases, both already suggested as metabolic hot spots of COVID-19 before (26). In sepsis and HIV-1 infection, the increased phenylalanine serum concentrations are linked to immune activation and increased cardiovascular event risk (27–29). Although the mechanisms behind this association are not well studied, it is in line with microvascular endothelial damage and higher coagulation risk characteristic to both: coronary heart disease and severe COVID-19 (29). Phenylalanine and tyrosine are catabolized to dopamine and epinephrine, and the latter has been employed in cardiac arrest as a result of cytokine storm, characteristic of severe COVID-19 patients (30).

We also performed a correlation analysis between the significant metabolites (Table 1) and available biochemical parameters (Table S1); however, none of the identified correlations reached statistical significance either in the acute or recovery stage.

The main limitation of our study is the relatively small sample size, which is mainly caused by the limited attainability of hospitalized COVID-19 patients during the pandemic. Nevertheless, we believe that this limitation is overcome by applying the longitudinal study design, which provides higher statistical power and minimizes the potential interference of individual-level confounding variables such as age and sex, meanwhile assuring the possibility to detect subject-specific effects. For technical reasons, we did not include lipidomics and untargeted metabolomics that could lead to a more comprehensive set of altered metabolites. Another aspect one may consider as the limitation is the consideration of serum samples obtained from independent subjects without any acute infection as control measures instead of measures of the same COVID-19 patients made before the onset of the infection, which would reflect the true serum metabolome modifying effects of the virus. During the particular study, the pre-infection serum samples were not collected, though they should be considered for similar future studies.

In conclusion, our study shows that metabolomic profiling provides novel insights into the pathogenesis of host-defense mechanisms and may be further applied for rapid biomarker discoveries in infectious diseases. These discoveries could show novel therapeutic strategies as indirect targets to fasten the recovery process after severe COVID-19. In addition, we believe that our data may contribute to the development of metabolomics-based personalized care strategies in COVID-19 patients comparable to already existing clinical approaches applied in health services such as newborn screening of inborn errors. To the best of our knowledge, this is the first longitudinal study covering metabolomic profiling during the recovery of severe COVID-19.

### Data availability.

Metabolomics data have been deposited to the EMBL-EBI MetaboLights database (DOI: https://doi.org/10.1093/nar/gkz1019, PMID:31691833) with the identifier MTBLS3852.
