# Supplementary material for: Amino Acid Metabolism is Significantly Altered at the Time of Admission in Hospital for Severe COVID-19 Patients: Findings from Longitudinal Targeted Metabolomics Analysis
Source: Microbiol Spectr. 2021 Dec 8;9(3):e00338-21. doi: 10.1128/spectrum.00338-21 (PMC8653833; doi:10.1128/spectrum.00338-21)
Supplement: SUPPLEMENTAL FILE 1 — Supplemental material. Download SPECTRUM00338-21_Supp_1_seq4.pdf, PDF file, 0.1 MB [file spectrum00338-21_supp_1_seq4.pdf]

Amino acid metabolism is significantly altered at the time of admission in hospital for severe COVID-19 patients: findings from longitudinal targeted metabolomics analysis

Laura Ansone & Monta Briviba, Ivars Silamikelis, Anna Terentjeva, Ingus Perkons, Liga Birzniece, Vita Rovite, Baiba Rozentale, Ludmila Viksna, Oksana Kolesova, Kristaps Klavins, Janis Klovins.

Text S1. Detailed Methods Description

Study Design and patient recruitment

In this study, we performed the longitudinal serum metabolome analysis in 33 Covid-19 patients with severe course of the disease (according to WHO Severity Definitions (1)). Patient recruitment was conducted in the major hospitals of Latvia (Riga East University Hospital and Vidzeme Hospital) during the acute phase of the disease. Written informed consent was obtained from every participant before their inclusion in the study, and the study protocol was approved by the Central Medical Ethics Committee of Latvia (No. 01-29.1.2/928). The peripheral blood samples for this study were collected in 1 to 3 days after admission to the hospital (acute phase) and 40.30±14.92 days later (recovery phase). Biochemical blood tests were performed in 0-48 hours from the hospitalization moment, clinical data were collected from the medical records of each hospital. Blood tests at the recovery phase were performed in certified clinical laboratories, which also ensured the sample collection. A longitudinal study design was chosen as the most suitable approach for evaluating metabolomics data in the COVID-19 cohort characterized with high inter-individual variability. Additionally, 39 individuals from the population with no acute infections at the time of application according to self-reported data were selected from the Genome Database of Latvian population (national biobank) and used as controls (Central Medical Ethics Committee of Latvia, approval No.1/19-04-05).

Sample processing

A set of 51 metabolites was selected to include representatives of metabolic pathways that are found to be consistently altered in viral infections (2) and compile a maximal number of technically

feasible analytes for which standard substances and isotopically labeled internal standards were available. Serum separation was performed by centrifuging peripheral blood samples collected in BD Vacutainer Blood Collection tubes at 4000 rpm, +4 C for 15 minutes. 800 μL of methanol was added to 200 μL of serum samples. Samples were vortexed for 10 seconds, shaken for 20 min at 450 rpm, and centrifuged for 10 min at 10000 g, 100 μL of sample extract was dried down using a centrifugal vacuum evaporator. Dry residues were reconstituted in 200 μL of methanol and 20 μL of the isotopically labeled internal standard mix was added. Afterward, samples were transferred to HPLC vials and used for LC-MS analysis.

LC-MS analysis

A Dionex 3000 HPLC system (Thermo Scientific) coupled with an Orbitrap Q Exactive (Thermo Scientific) mass spectrometer was used for the LC-MS analysis. The chromatographic separation was carried out on an ACQUITY UPLC BEH Amide, 1.7 μm, 2.1x100 mm analytical column (Waters) equipped with a VanGuard: BEH C18, 2.1x5 mm pre-column (Waters). The column was maintained at a temperature of 40 °C and the sample injection volume was 2 μL. The mobile phase consisted of phase A - 0.15% formic acid (v/v) in water and phase B - 0.15% formic acid (v/v) in 85% acetonitrile (v/v) with 10 mM ammonium formate. The gradient elution with a flow rate of 0.4 mL/min was performed for a total analysis time of 17 min. The Orbitrap Q Exactive (Thermo Scientific) mass spectrometer was operated in a positive electrospray ionization mode, spray voltage 3.5 kV, aux gas heater temperature 400 °C, capillary temperature 350 °C, aux gas flow rate 12, sheat gas flow rate 50. The metabolites of interest were analyzed using a full MS scan mode, scan range m/z 50 to 400, resolution 35000, AGC target 1e6, maximum IT 50 ms. The Trace Finder 4.1 software (Thermo Scientific) was used for data processing. A seven-point linear calibration curve with internal standardization and 1/x weighing was constructed for the quantification of the metabolites.

Data analysis

Longitudinal analysis. Data matrix containing time point information and quantified metabolite concentrations (μmol/L) that were above Level Of Detection (LOD) in more than 50% of the samples were uploaded in web-based, free software Metaboanalyst, version 5.0 (https://www.metaboanalyst.ca/). The uploaded data file contained 66 samples (33 pairs) by 51 compounds data matrix. A total of 67 (3.3%) missing values were detected, missing values were replaced by 1/5 of the min positive values of their corresponding variables. One of the samples was identified as an outlier and both samples from that patient were excluded from further analysis. Normalization by sum, log transformation, and Pareto scaling was performed. The univariate analysis included paired Fold Change analysis to evaluate the levels and direction of change of metabolites and paired t-test to identify significant targeted metabolites between acute and recovery phases.

Case-control analysis. Data matrix was uploaded in Metaboanalyst, version 5.0, it consisted of quantified metabolite concentrations for the same 31 compounds used for longitudinal analysis. For the comparative purpose, the samples were considered as 3 groups - Acute COVID-19, Recovery phase, and Non-COVID controls. A total of 128 (3.9%) missing values were detected, missing values were replaced by 1/5 of the min positive values of their corresponding variables. The same normalization, transformation, and scaling methods were applied as described before.

The heatmap in Figure 1A was generated with normalized data for 51 features, using Ward Clustering Algorithm and Euclidean Distance Measure. Principal Component Analysis (PCA) was performed to evaluate the distribution of samples within the 95% confidence region in all three groups (Figure 1B). Principal component analysis and visualization were performed with Python libraries scikit-learn 1.0.1, pandas 1.2.4, matplotlib 1.2.4, numpy 1.20.3. Prior to analysis, samples with missing values were excluded and subsequent metabolite value standardization was performed.

The pathway analysis was also done in Metaboanalyst 5.0 (https://www.metaboanalyst.ca/) for longitudinal samples only, quantified concentrations (μmol/L) were uploaded, data were normalized

by sum and log transformation, Pareto scaling was applied. Pathway analysis was visualized with Scatter plot (Figure 1C), Enrichment method was set to Global Test, and as Reference pathway library was selected *Homo Sapiens* (KEGG). Figures 1A, C, D were drawn via metaboanalyst software v 5.0 (https://www.metaboanalyst.ca/). For average, percentage, and standard deviation calculations *MS Excel* was used.

Spearman's correlation coefficients between the significant metabolites listed in Table 1 and available biochemical parameters obtained from regular blood tests (Table S1), including the level of C reactive protein and D-dimers were calculated using the corr.test function implemented in the psych package of R (v.4.0.3). in COVID-19 patient dataset for each time point of blood collection (acute and recovery phase) separately.

References:

1.World Health Organization. (2021). COVID-19 clinical management: living guidance, 25 January 2021. World Health Organization.

2.Sanchez, E. L. & Lagunoff, M. Viral activation of cellular metabolism. Virology 479–480, 609–618.(2015).

Table S1. Characteristics of the study participants.

**Variable,** mean (SD) or n (%)	**COVID-19 patients**	**Population controls**
Male/ Female (n, %)	16 (48.5 %)/17 (51.5 %)	16 (41.0 %) / 23 (59.0 %)
Age, average ± SD (years ± SD)	58.52 (16.26)	53.46 (14.75)
BMI, average (kg/m2 ± SD)	32.66 (24.04)	28.56 (6.45)
Time in Hospital (days ± SD)	11.67 (5.22)	-
	**Comorbidities***	
**Number of patients with comorbidities, (yes/no)**	21 (63.6 %)/12 (36.4%)	23 (59.0%)/16 (41.0%)
Hypertension (n, %)	15 (45.5%)	8 (20.5%)
Type 2 Diabetes Mellitus (n, %)	3 (9.1%)	3 (7.7%)
Respiratory system (n, %)	1 (3.3%)	0
Other cardiovascular disease (n, %)	7 (21.2%)	12 (30.8%)
Oncological (n, %)	1 (3.3%)	3 (7.7%)
Other	10 (30.3 %)	13 (33.3 %)
	**Clinical measurements****	
**Average (SD)**	**Acute COVID-19**	**Recovery phase**
Leukocytes (µL)	5.52 (1.66)	5.86 (1.28)
Hemoglobin (g/dL)	13.74 (1.27)	13.78 (1.18)
Hematocrit (%)	41.41 (3.56)	42.06 (3.72)
Platelets (µL)	202.94 (65.26)	273.86 (63.55)
Neutrophils (µL)	2.18 (1.78)	2.99 (0.74)
Lymphocytes (µL)	0.64 (0.56)	2.09 (0.63)
Monocytes (µL)	0.24 (0.20)	0.56 (0.20)
Eosinophils (µL)	0.02 (0.04)	0.19 (0.11)
ALT (U/l)	33.78 (25.41)	33.07 (27.14)
AST (U/l)	41.94 (27.24)	27.79 (16.61)
GGT (U/l)	79.09 (86.25)	48.41 (48.94)
Bilirubin (µmol/l)	9.19 (5.54)	13.60 (5.15)

LDH (U/L)	312.46 (177.19)	214.45 (37.06)
Potassium (mmol/L)	4.10 (0.42)	-
Sodium (mmol/L)	134.67 (4.13)	-
Creatinine (µmol/L)	80.47 (28.66)	68.52 (17.04)
CRO (mg/L)	56.98 (58.43)	3.90 (6.14)
D-dimer (mg/ml)	83.89 (256.23)	0.64 (0.62)
Ferritin (mg/L)	720.55 (1149.92)	-
Troponin T (pg/mL)	13.11 (10.33)	-

**Comorbidities for population controls were self-reported. ** Clinical measurements in Acute COVID-19 phase were performed at in-house hospital clinical laboratory, but Recovery phase measurements in the largest local network of certified clinical laboratories outside hospitals.SD: standard deviation; BMI: body mass index; ALT: alanina aminotransferase; AST: aspartate aminotransferase; GGT: gamma-glutamyl transferase; LDH: lactate dehydrogenase; CRO: C-reactive protein.*

Table S3. Significant pathways based on enrichment procedures.

**Pathway Name**	**False discovery rate**	**Impact**
Phenylalanine, tyrosine and tryptophan biosynthesis	7.14E-07	1.00
D-Glutamine and D-glutamate metabolism	9.50E-05	0.50
Arginine biosynthesis	3.76E-06	0.48
Taurine and hypotaurine metabolism	2.05E-02	0.43
Arginine and proline metabolism	2.95E-07	0.39
Phenylalanine metabolism	7.14E-07	0.36
Alanine, aspartate and glutamate metabolism	4.09E-06	0.31
Tryptophan metabolism	4.09E-06	0.24
Histidine metabolism	2.89E-02	0.22
Tyrosine metabolism	8.84E-04	0.14
Cysteine and methionine metabolism	3.41E-02	0.10
Glutathione metabolism	2.86E-03	0.02
Primary bile acid biosynthesis	2.05E-02	0.01
